# Editorial: The applications of AI techniques in medical data processing

**DOI:** 10.3389/frai.2026.1794193

**Published:** 2026-02-26

**Authors:** Pengfei Zhang

**Affiliations:** The School of Intelligent Medicine, Chengdu University of Traditional Chinese Medicine, Chengdu, China

**Keywords:** medical AI, Machine Learning, medical data processing, clinical decision support, multimodal learning, explainable AI (XAI), Large Language Models (LLMs), healthcare

The digital transformation of healthcare has ushered in an era where data is no longer merely a record of clinical encounters, but a dynamic asset capable of driving precision medicine. This Research Topic, “*The Applications of AI Techniques in Medical Data Processing*,” explores the diverse ways in which Artificial Intelligence (AI) and Machine Learning (ML) are being leveraged to refine diagnostics, predict clinical outcomes, and optimize the infrastructure of modern medicine. This Research Topic of 21 articles showcases a multidisciplinary effort to bridge the gap between complex algorithmic development and practical clinical application.

## 1 Predictive modeling and clinical decision support

A central theme of this Research Topic is the utilization of ML to identify high-risk patients across diverse clinical scenarios. In acute care, studies on urosepsis emphasize the necessity of interpretability. By employing XGBoost and SHapley Additive exPlans (SHAP), Wei et al. identifies high-risk individuals with an AUC of 0.904. This focus on clinical utility is supported by Byberg and Crimi, which argues that prognostic accuracy must be matched by robust organizational readiness.

Predictive efficacy is further demonstrated in cardiovascular and respiratory conditions. Ghandour et al. achieves an AUC of 0.76 by combining patient baseline characteristics with viscoelastic testing. Similarly, Su et al. utilizes a logistic regression-based nomogram to reach an accuracy of 90.13%. For heart transplantation, a meta-analysis in Mohammadi et al. identifies CatBoost as the superior algorithm while cautioning against high risks of bias in current modeling flows. In infectious disease screening, Ihenetu et al. finds that random forest classifiers significantly outperform traditional logistic regression for rapid symptom-based screening.

## 2 Specialized diagnostics, medical imaging, and genomic insights

Advanced imaging and multi-modal architectures represent a primary frontier in this Research Topic. Ciusdel et al. introduces a novel framework that enhances accuracy across 135 different segmentation tasks by leveraging support-set pre-selection. More targeted approaches include Dardouri, which achieves a 99.68% accuracy in stage-specific AD detection.

The integration of metadata is also crucial; Das et al. highlights how cross-attention fusion between dermatoscopic images and clinical attributes outperforms image-only baselines. In ophthalmology, Barry and Wang utilizes 1D-CNNs to predict surgical failure with an AUROC of 76.4%. Furthermore, genomic analysis is advanced by Wang et al., which employs Kolmogorov-Arnold Networks (KAN) to map complex nonlinear relationships between genetic features like DEPDC5 and LGI1 and disease status.

## 3 Chronic care, mental health, and the evolving role of LLMs

AI is deeply integrated into chronic disease and mental health management. Jin and Halili reveals that subjective perceptions, such as life satisfaction, contribute more to prediction than traditional biomedical indicators. For pain management, Visibelli et al. utilizes random forest classifiers to achieve 80% accuracy in predicting treatment adherence, identifying high THC dosage as a primary risk factor for dropout.

The role of Large Language Models (LLMs) presents a critical dichotomy. Yoon et al. shows a promising 51.3% overlap between AI and expert acupoint selection, suggesting educational potential. Conversely, ElSayed and Updegrove reveals that generalist models like Claude 3.5 fail significantly in specialized visual tasks, with DeepSeek R1 achieving only 44% accuracy on orthopedic diagrams.

## 4 Foundations: infrastructure, real-time processing, and ethics

Scaling medical AI requires specialized foundational systems. Lyu et al. notes that while specialized data warehouses excel in decision support, they face unique scalability challenges compared to general-purpose architectures. This infrastructure gap is further emphasized in Yang et al., which identifies information system effectiveness as the most critical predictor of pharmacovigilance success.

Efficiency and explainability remain paramount. Gobin et al. introduces ELISE as a tool specifically designed for regulatory and clinical traceability. For real-time applications, Haque et al. demonstrates how GA-GAN-XAI frameworks can achieve 99.49% accuracy with minimal latency. Furthermore, Yu and Zhu proposes Dynamic Medical Graph Frameworks (DMGF) to model temporal relationships in multi-modal neuroscience data.

Crucially, the ethical deployment of these tools must address technical vulnerabilities. Rai et al. provides a critical observation: class imbalance in clinical datasets significantly undermines the consistency of LIME and SHAP explanations, highlighting a potential risk for trustworthy AI deployment.

## 5 Conclusion and future outlook

This Research Topic illustrates that the application of AI in medical data processing is entering a more mature and critical stage of development. The emphasis is gradually shifting beyond isolated gains in predictive accuracy toward deeper inter-modal integration, ethical transparency, and the readiness of supporting clinical and computational infrastructures. As these technologies continue to evolve, the effective integration of expert human oversight with high-performing and interpretable algorithms will become a cornerstone for building safer, more reliable, and more efficient healthcare systems.

The 21 contributions in this Research Topic collectively signal that medical AI is transitioning from “algorithmic novelty” to “clinical maturity.” By synthesizing the findings of this diverse Research Topic, four critical forward-looking observations for the field are summarized as follows.

1) The fragility of interpretability in skewed data: a pivotal insight from our Research Topic is the sensitivity of XAI tools like LIME and SHAP to class imbalance in clinical datasets. Future research must shift from merely applying *post-hoc* explanations to developing distribution-aware interpretability frameworks that remain consistent across varying disease prevalences, ensuring that AI-driven clinical reasoning does not misguide physicians in rare-disease scenarios.
2) The transition from generalist LLMs to specialized vision-language models: our findings contrast the promising diagnostic support of LLMs in textual domains with their stark failure in visual medical classification (e.g., orthopedic Walch classifications). This suggests that the future of medical AI lies in domain-specific foundation models that integrate expert medical-grade visual reasoning with textual knowledge, rather than reliance on broadly trained generalist architectures.
3) Infrastructure as the primary determinant of scalability: the success of the “One Body, Two Wings” pharmacovigilance model and the insights from data warehousing reviews highlight that the bottleneck of clinical AI is increasingly structural rather than computational. Future investments must prioritize AI-native healthcare IT ecosystems that balance specialized analytical power (specialized warehouses) with the interoperability required for large-scale clinical impact.
4) Hyper-multimodal and temporal synergy: the move toward dynamic medical graphs and in-context cross-attention for segmentation demonstrates that the standard for precision diagnostics is shifting toward temporal integration. Integrating longitudinal patient patterns with high-dimensional multi-modal data (genomics, clinical metrics, and imaging) will be the cornerstone for realizing truly personalized, data-driven healthcare.

